# How to Quantitatively Balance a Total Knee? A Surgical Algorithm to Assure Balance and Control Alignment

**DOI:** 10.3390/s21030700

**Published:** 2021-01-20

**Authors:** Ryan E. Moore, Michael A. Conditt, Martin W. Roche, Matthias A. Verstraete

**Affiliations:** 1 Coon Joint Replacement Institute: Adventist Health, St. Helena, CA 94574, USA; rymoore2009@gmail.com; 2 OrthoSensor Inc., Dania Beach, FL 33004, USA; michael.conditt@orthosensor.com; 3 Holy Cross Hospital, Fort Lauderdale, FL 33308, USA; martin@mroche.com; 4 Department of Structure and Repair, Ghent University, 9000 Ghent, Belgium

**Keywords:** balancing, surgical corrections, soft tissue releases, mechanical alignment, total knee arthroplasty

## Abstract

To achieve a balanced total knee, various surgical corrections can be performed, while intra-operative sensors and surgical navigation provide quantitative, patient-specific feedback. To understand the impact of these corrections, this paper evaluates the quantitative impact of both soft tissue releases and bone recuts on knee balance and overall limb alignment. This was achieved by statistically analyzing the alignment and load readings before and after each surgical correction performed on 479 consecutive primary total knees. An average of three surgical corrections were required following the initial bone cuts to achieve a well aligned, balanced total knee. Various surgical corrections, such as an arcuate release or increasing the tibial polyethylene insert thickness, significantly affected the maximum terminal extension. The coronal alignment was significantly impacted by pie-crusting the MCL, adding varus to the tibia, or releasing the arcuate ligament or popliteus tendon. Each surgical correction also had a specific impact on the intra-articular loads in flexion and/or extension. A surgical algorithm is presented that helps achieve a well-balanced knee while maintaining the sagittal and coronal alignment within the desired boundaries. This analysis additionally indicated the significant effect that soft tissue adjustments can have on the limb alignment in both anatomical planes.

## 1. Introduction

Knee stability after total knee arthroplasty (TKA) is of primary importance to avoid early revision surgery [1]. Recent studies indicate superior patient-reported outcomes following TKA cases that have been quantitatively balanced, including increased patient satisfaction [2,3] and improved forgotten joint scores [3], as well as fewer soft tissue-related post-operative complications, such as manipulations under anesthesia [4]. Meanwhile, the surgical technique to obtain a balanced knee remains challenging, as restoring near-neutral, functional, or kinematic alignment may require substantial soft tissue adjustments or bone recuts. While it has been shown that without quantitative feedback during balancing, surgeons are only able to achieve a balanced knee approximately 50% of the time [3,5,6], technology to assist balancing has proven to effectively and consistently balance a knee through a range of motion following an acceptable learning curve [7,8,9]. In fact, it has been shown that quantitative sensor feedback is instrumental in achieving balance [5,10,11]. The political science writer Philip E. Tetlock is quoted in “Superforecasting: The Art and Science of Prediction” as saying “If you don’t get feedback, your confidence grows much faster than your accuracy”.

Feedback is necessary but not sufficient to solve the balancing problem, however, as the knee is a multi-dimensional, overdetermined system with redundancy, creating a complex soft tissue envelope that engages differently at different flexion angles. Clearly, adjustments to any soft tissues may have a complex, non-linear effects on the stabilizing nature of the soft tissue envelope, and bone cuts that change the joint line (in any of the three dimensions, including axial rotation) can also change the soft tissue contributions through flexion. Basic cadaveric work has shown the effect of surgical corrections [12] but has always been limited in terms of number of specimens due to the inherent limitations of cadaveric testing. Though limited, these data show that a knee can go from an imbalanced state to a balanced state with adjustments within only 2 degrees or 2 mm. Small clinical series (between 100 and 200 patients) have also shown that between two and three corrections, with as many as eight, are necessary to achieve balance [13,14]. A more recent study has shown the importance of sensor technology in supporting multiple intra-operative decisions aimed at reproducing desired kinematic targets [15]. While all of this work has led to a general surgical algorithm for addressing imbalance based on its asymmetry and where it occurs (flexion, extension, or both) [13], the actual effects of each sequential correction on the loads are still undocumented.

The hypothesis of this study is that a consistent surgical algorithm can be formed by examining a large series of successfully quantitatively balanced total knees. To date, there have been more than 30,000 procedures performed with quantitative balancing. While the meticulous documentation of every case is not feasible, this paper examines every sequential step taken by one experienced surgeon to achieve a balanced knee in a large series of cases, with the goal of understanding how each surgical correction affects both balance—as measured by intercompartmental loads—as well as limb alignment.

## 2. Materials and Methods

### 2.1. Clinical Data Collection

Within an IRB-approved study, all primary total knee surgeries between January 2017 and August 2018 performed by a single fellowship-trained surgeon were included in this study, resulting in a total of 479 consecutive cases. A single, posterior stabilized implant was used for all cases (Triathlon, Stryker), with the surgeon targeting neutral mechanical alignment and a quantitatively balanced knee. In all cases, surgical navigation was used during surgery (Stryker OrthoMap, Mahwah, NJ, USA), which tracks the three-dimensional relative motion of the femur and tibia. This navigation system allows the quantitative assessment of the coronal and sagittal alignment, with the goal of neutral mechanical alignment in the coronal plane and achieving full extension in the sagittal plane. To evaluate the state of knee balance during surgery, smart tibial trial components were used intraoperatively (VERASENSE, OrthoSensor, Fort Lauderdale, FL, USA). These trial components assessed the medial and lateral tibiofemoral loads through a range of motion. For the purposes of this study, loads in the medial and lateral compartment were collected at 10 and 90 degree of flexion, with the balance being defined as a mediolateral load differential of less than 15 lbf at both 10 and 90 degrees of flexion, with no load exceeding 40 lbf or less than 10 lbf, as previously reported [13]. The knee was held in a neutral position during load measurements, with no external axial or varus/valgus loads applied. If necessary to achieve a balanced knee, surgical corrections were performed, as discussed in the next paragraph. Both the sensor feedback, indicating balance, and the navigation feedback, indicating alignment, were collected before and after each surgical correction. 

### 2.2. Surgical Corrections

**Figure 1:**
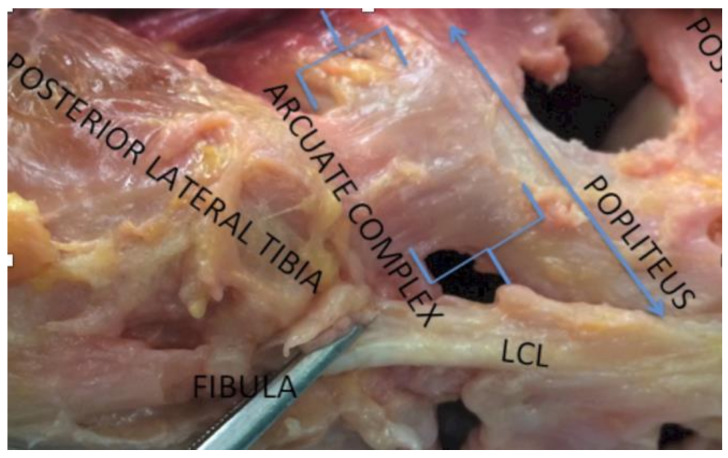
Posterolateral corner of the knee showing the arcuate ligament.

The surgical corrections that were used to achieve a balanced knee consisted of soft tissue adjustments, bone recuts, and changes in tibial polyethylene insert thickness. A total of five possible soft tissue adjustments were documented during this study:

- *MCL pie-crusting*: The (anterior or posterior) tight bands of the medial collateral ligament (MCL) were palpated and punctured using an 18-gauge needle [16,17]. The number of punctures varied on a case by case basis and was not documented during this study.
- *Arcuate release*: Located in the posterolateral corner, the medial limb of the arcuate complex curves over the popliteus muscle to join with the oblique popliteal ligament, while the lateral limb ascends to blend with the capsule near the lateral gastrocnemius muscle (Figure 1). Both limbs of the arcuate complex were incised at the joint line with the knee in extension using curved scissors.
- *Popliteus release*: Located in the posterolateral corner, the popliteus connects the posterolateral femoral condyle to the popliteus muscle. During a release, this ligamentous structure is being resected with a 11- or 15-blade.
- *Posterior capsule release*: The attachment of the posterior capsule to the posterior distal femur was subperiosteally released using electrocautery and a Cobb elevator.
  *ITB release*: The iliotibial band (ITB) is located on the lateral side of the tibia was incised at the joint line using curved scissors.

In addition to soft tissue adjustments, knee imbalance was often addressed by revisiting the initial bone resections. Recuts were performed at the level of both the tibia and the femur and grouped as either a tibia or femur recut. The initial tibial cut was performed perpendicular to the tibial mechanical axis, as guided by surgical navigation. A tibia recut therefore typically resulted in increased tibial varus alignment; no tibial valgus alignment was accepted. For the femur, various types of recuts were performed with respect to the distal cut (and associated chamfer cuts) to 1) increase the varus angle of the distal femur by resecting additional bone from the medial distal femur and then recutting the femur with a 4 in 1 cutting block or 2) to elevate the joint line without changing the varus angle by resecting bone equally from both the medial and lateral distal femur and then recutting the chamfers; femoral rotation was not altered while balancing the knee. A final possible surgical correction consisted of increasing the poly insert thickness; for the implant system under investigation, this represented a 2 mm increase.

### 2.3. Statistical Analysis

Intra-operatively, the load and alignment data prior to and following a surgical correction were tabulated, allowing the quantification of the change in load and alignment associated with each maneuver. Often, several surgical corrections were performed during the same procedure, thus potentially creating confounding factors. To understand the effect of individual surgical corrections, these tabulated changes in the load and alignment parameters were analyzed using linear mixed models. The various types of corrections were seen as categorical variables, while the load and alignment parameters were seen as continuous outcome variables. These models additionally account for patient-to-patient variability by considering the patient as a random effect. An additional advantage of these models is their ability to deal with missing values as well as to account for repeated measures, such as when several surgical corrections were performed for the same patient.

## 3. Results

### 3.1. Patient Population

Our population was 479 consecutive cases consisting of 49% male and 51% female patients with a mean age at the time of surgery of 68.6 years (range: 39 to 96). A total of 21 knees had missing data with insufficient documentation of the load and alignment data, leading to a total of 1391 recorded surgical corrections.

### 3.2. Surgical Corrections

**Figure 2:**
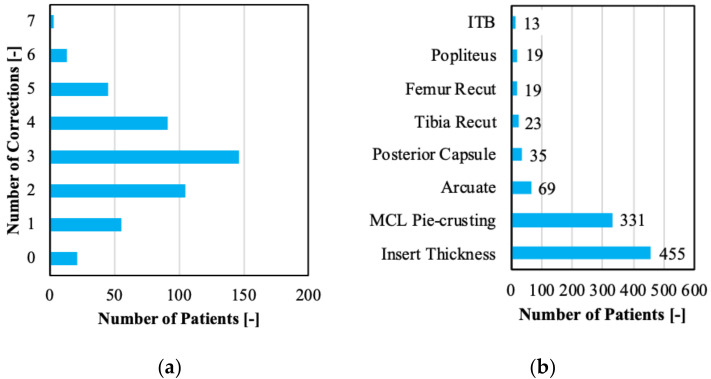
(**a**) Evaluation of number of surgical corrections per patient and (**b)** type of surgical corrections encountered in the clinical dataset.

An average of 3.03 surgical corrections were required per patient to achieve a well-balanced and aligned knee. The number of corrections varied between zero corrections for some and up to seven corrections for other cases (Figure 2a). Focusing on the specific surgical corrections, pie-crusting the MCL and increasing the poly insert thickness were the most common interventions (Figure 2b).

### 3.3. Effects on Alignment

**Figure 3:**
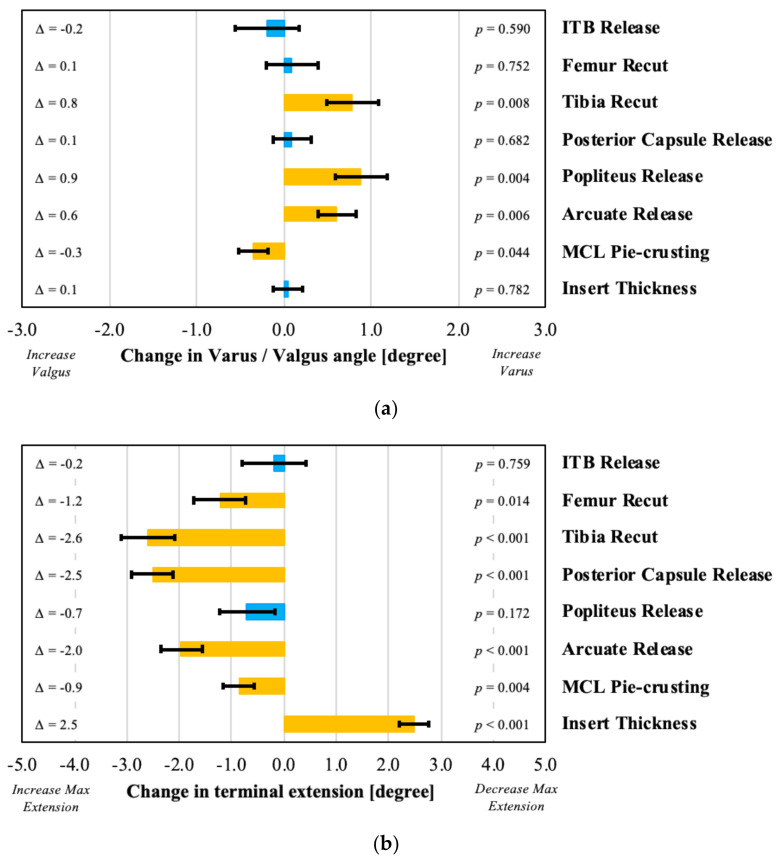
Evaluation of effect of various surgical decisions on (**a**) coronal (negative represents varus and positive represents valgus) and (**b**) sagittal alignment in extension (positive represents a decrease in terminal extension), with orange bars indicating statistically significant effects.

The effects of the various surgical corrections are seen both in terms of the intra-articular loads (as discussed in the next section) and in terms of the coronal and sagittal alignment. This section focuses on the alignment parameters, demonstrating that lateral soft tissue releases, such as an arcuate or popliteus release, significantly increased the overall limb varus alignment (Figure 3a). A tibial varus recut also resulted in increased varus alignment. In contrast, the pie-crusting of the MCL resulted in added valgus alignment. Surgical corrections also affected the sagittal alignment, with an increase in insert thickness of 2 mm, decreasing the terminal extension by 2.5 degrees on average (*p* = 0.0002; Figure 3b). All other surgical corrections, apart from an ITB and popliteus release, increased the maximum extension angle.

### 3.4. Effects on Intra-Articular Loads

**Figure 4:**
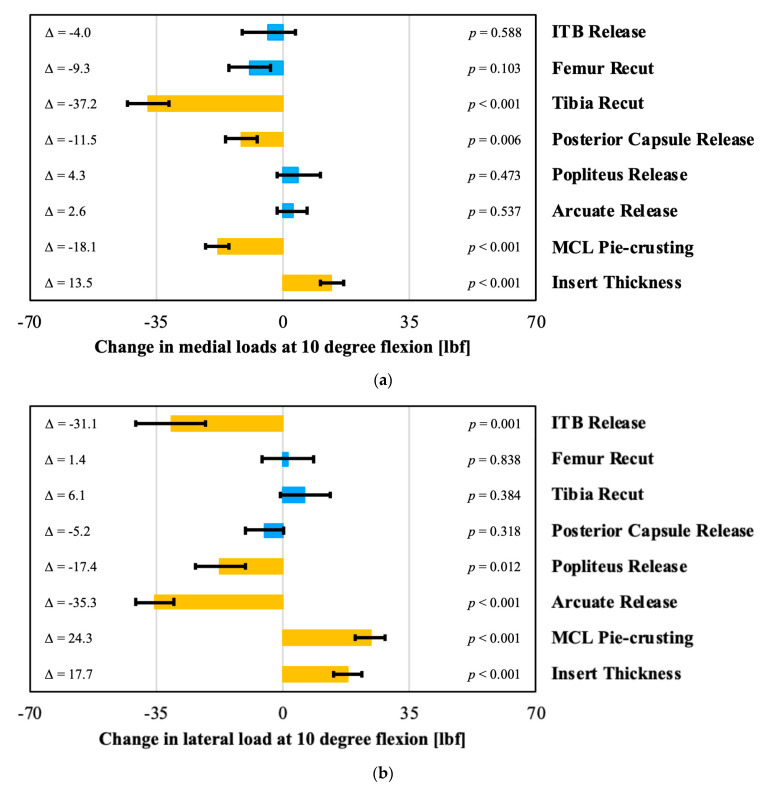
Evaluation of the effect of surgical correction on (**a**) medial (**b**) and lateral loads in 10 degree of flexion, with orange bars indicating statistically significant effects. Each change is the post-correction value minus the pre-correction value.

**Figure 5:**
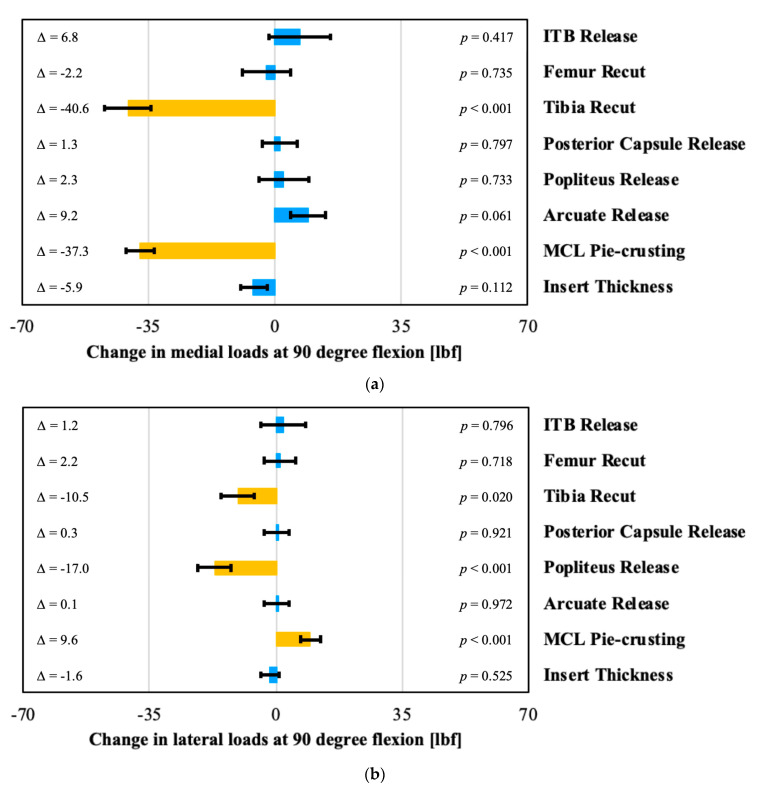
Evaluation of the effect of surgical correction on (**a**) medial (**b**) and lateral loads in 90 degrees of flexion, with orange bars indicating statistically significant effects. Each change is the post-correction value minus the pre-correction value.

The effects of the surgical corrections on the intra-articular loads at 10 and 90 degrees flexion are shown in Figure 4 and Figure 5. A tibial varus recut, a posterior capsule release, and pie-crusting the MCL all significantly decreased the medial loads at 10 degrees. In contrast, the medial loads increased significantly with increased insert thickness (Figure 4a). On the lateral side in 10 degrees flexion, the loads decreased significantly after an ITB, popliteus, or arcuate release (Figure 4b). In contrast, these loads increased significantly after pie-crusting the medial collateral ligament or increasing the insert thickness.

In flexion, the medial loads decreased significantly after a tibial recut and MCL pie-crusting (Figure 5a). On the lateral side, this pie-crusting increased the loads significantly. In contrast, the loads in flexion decrease laterally after a popliteus release and re-cutting the tibia (Figure 5b).

## 4. Discussion

**Figure 6:**
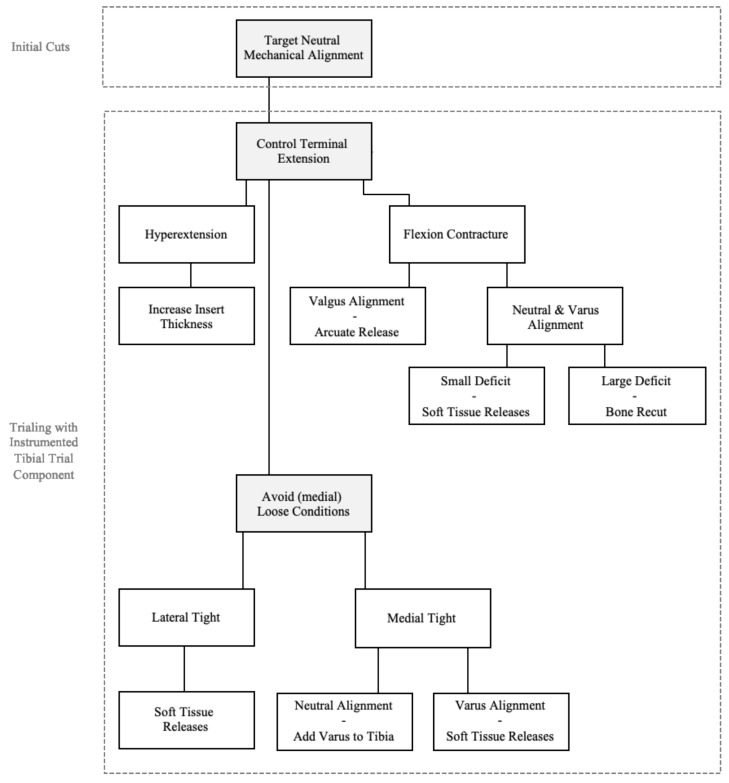
Surgical decision process to assure satisfactory alignment and soft tissue balance while balancing a total knee during the trialing phase.

To balance a total knee, various surgical corrections can be performed depending on the alignment philosophy and balance targets. Since the introduction of intra-operative sensor technology, the latter has become quantifiable, whereas the former can be quantified intra-operatively using surgical navigation or robotics technology. In this paper, changes in the intra-articular loads and alignment parameters have been quantitatively assessed following various surgical corrections, thereby providing insight into the motivation for selecting various maneuvers. This results in a structured approach to achieve a quantitatively balanced knee that concurrently meets the surgeon’s defined alignment targets. This approach has schematically been presented in Figure 6.

While performing the initial femoral and tibial cuts, a neutral overall mechanical limb alignment is targeted with a neutral tibial cut. During the subsequent trialing phase, quantitative feedback is collected from the surgical navigation system and the instrumented tibial trial component. Terminal extension is first assured, whereby a soft tissue release of the posterior capsule is considered for small extension deficits as it does not impact the mediolateral load balance. Larger extension deficits are typically addressed through bone recuts. As such, a recut of the femur is considered when compartmental loads in flexion are appropriate and acceptable, hence increasing the extension space without affecting the knee balance in flexion. Analogously, a tibial recut is considered when the loads are excessive in both flexion and extension and a flexion contracture exists in order to simultaneously affect the knee in both flexion and extension. One exception to this approach is when considering a flexion contracture in a valgus knee, where an arcuate release has the potential to address both the flexion contracture as well as elevated loads in the lateral compartment. In contrast to a flexion contracture, hyperextension is addressed by increasing the poly insert thickness, as also indicated by the data presented in this paper.

Following the assessment and necessary adjustments to achieve terminal extension in the sagittal plane, the load sensor readings at 10 and 90 degrees become of primary importance, while feedback from the navigation system is used concurrently to understand the overall coronal alignment. Loose conditions should first be addressed by increasing the poly insert thickness. This is particularly relevant when observed medially, as a stable medial column through the range of motion should uncompromisingly be the primary balance target.

Subsequently, various tightness conditions can be observed that lead to various compensatory strategies. Lateral tightness is addressed through soft tissue releases to avoid overall limb valgus as laterally tight conditions typically occur in knees with overall limb valgus. Therefore, lateral soft tissue releases, particularly the arcuate release, have the ability to both decrease lateral loads and bring the overall valgus alignment towards neutral alignment. Different soft tissues will be targeted depending on whether the imbalance is observed in flexion (90 degree) and/or extension (10 degree). An isolated lateral extension imbalance is typically achieved through an arcuate release and/or a release of the iliotibial band. Both, indeed, result in a controlled and significant reduction in the lateral loads in extension. In contrast, an isolated flexion imbalance is addressed through a release of the popliteus. The statistical analysis confirmed a significant decrease in the lateral loads in flexion following a popliteus release but additionally indicated the potential decrease in the lateral loads in extension. If a knee is laterally tight through the range of motion, a combination of releases as discussed above is considered.

When it comes to medial tightness, isolated extension tightness is addressed either through pie-crusting the medial collateral ligament or cutting additional varus in the femur. The former is considered when the overall limb alignment is outside the ±3 degree window from neutral. Medial tightness in flexion is also addressed through MCL pie-crusting. When the knee is tight medially in both flexion and extension and the overall limb alignment is varus, preference is given to MCL pie-crusting, which decreases medial loads while also correcting the varus limb alignment towards neutral. When the knee is tight medially in both flexion and extension and limb alignment is neutral or valgus, then a varus tibial recut is performed as MCL pie-crusting could result in resultant valgus alignment. Whereas this study did not differentiate between releasing the anterior versus posterior bands of the medial collateral ligament during data collection, it is important in the surgical technique to realize that the former primarily affect the flexion balance whereas the latter affect the extension balance [18,19]. Caution must thus be taken to not overly release the anterior structures of the MCL in a knee that is medially tight in extension, thereby avoiding the loosening of the flexion space. Analogous remarks can be made for releasing the posterior bands in a medial tight knee in flexion. When a knee is medially tight through the range of motion, the decision to cut additional varus in the tibia or pie-crust the MCL is again driven by the overall limb alignment.

This paper thus shows the complex interaction between alignment in both the sagittal and coronal planes and the role of soft tissues to balance a knee. Whereas the surgical algorithm presented here stresses the relevance of the alignment condition in selecting the appropriate surgical decisions when balancing a total knee, this paper also indicates the implications of soft tissue adjustments to the limb alignment. More specifically, it is seen that lateral releases that impact the loads in extension have the potential to significantly reduce overall valgus alignment Analogously, MCL releases increase valgus alignment. It is thus not surprising to see that a medial release (i.e., MCL pie-crusting) increases the lateral loads as the alignment changes, subsequently engaging the lateral compartment.

The surgical algorithm presented in this paper is in good agreement with the algorithm presented by Gustke et al. [13] using the same implant design with a few important distinctions. Their work was, however, based on a smaller series of 129 patients with a mixture of cruciate retaining (81%) and posterior stabilized (19%) cases, while this study included PS knees exclusively. More importantly, the algorithm presented by Gustke does not include any assessment or correction for overall limb alignment. Thus, the current algorithm may be seen as an evolution of the existing algorithm, with the additional goal of controlling limb alignment in both the sagittal and coronal planes.

This research also has some important limitations. First, this paper presents the results of a single surgeon series. Whereas the volume of cases allowed statistical interpretation, it is clear that this presents a potential bias in terms of alignment strategy (i.e., strict mechanical alignment) as well as implant design. The latter is particularly relevant, as only posterior stabilized implants were used. Retaining the posterior cruciate ligament likely alters the surgical algorithm as the PCL engages in flexion and might indicate other surgical corrections when imbalance is encountered in flexion. A second shortcoming is related to the granularity of the collected data. This presents for instance limitations on the interpretation of the MCL data, since no differentiation was made during data collection between various bands of the medical collateral ligament as it relates to balancing in flexion versus extension. A similar limitation is seen in the interpretation of the femoral recut data. Since this is not a common surgical correction, various types of femoral recuts (varus or proximalization of the implant) had been grouped as a single category, resulting in significant findings only related to addressing extension deficits and not in terms of load changes. Larger, more specific data collection could potentially address this. Another limitation is that our study does not include clinical outcome measures based on this approach to balance and align the knee, which would provide important clinical validation based on patient functional results. This will be part of our future research.

## 5. Conclusions

Based on a clinical series of 479 primary total knees, this paper presents a surgical algorithm to quantitively balance a knee while also ensuring appropriate terminal extension and keeping the coronal alignment within mechanically neutral boundaries, specifically avoiding valgus alignment and allowing for a maximum of three-degree varus overall alignment. Through the statistical analysis of various surgical corrections performed during these clinical cases, this algorithm is consistently supported by the quantified effects of various bone recuts and soft tissue adjustments. This quantification relates both to the intra-articular loads, as measured using an instrumented tibial trial, and limb alignment in the sagittal and coronal planes, as measured using surgical navigation, notwithstanding the need to recognize the variability in both the ligament behavior as well as the surgical technique.

This work additionally amplifies the relevance of these alignment parameters during the surgical decision process, while also elucidating the changes in intra-articular loads linked with specific surgical corrections.

## Data Availability

Restrictions apply to the availability of the data. Data was obtained from St Helena Hospital and are available from the authors with permission from St Helena Hospital.

## References

[1] Leone W., Geller J., Chow J., Branovacki G., Do J.M., Meere P. (2016). Using Sensors to Evaluate Revision TKA: Treating the “Looks Good; Feels Bad” Knee. EC Orthop..

[2] Gustke K.A., Golladay G.J., Roche M.W., Elson L.C., Anderson C.R. (2014). A New Method for Defining Balance. J. Arthroplast..

[3] Golladay G.J., Bradbury T.L., Gordon A.C., Fernandez-Madrid I.J., Krebs V.E., Patel P.D., Suarez J.C., Higuera C.A., Barsoum W.K. (2019). Are Patients More Satisfied with a Balanced Total Knee Arthroplasty?. J. Arthroplast..

[4] Geller J.A., Lakra A., Murtaugh T. (2017). The Use of Electronic Sensor Device to Augment Ligament Balancing Leads to a Lower Rate of Arthrofibrosis after Total Knee Arthroplasty. J. Arthroplast..

[5] Elmallah R.K., Mistry J.B., Cherian J.J., Chughtai M., Bhave A., Roche M.W., Mont M.A. (2016). Can We Really “Feel” a Balanced Total Knee Arthroplasty?. J. Arthroplast..

[6] MacDessi S.J., Gharaibeh M.A., Harris I.A. (2019). How Accurately Can Soft Tissue Balance Be Determined in Total Knee Arthroplasty?. J. Arthroplast..

[7] Gharaibeh M.A., Chen D.B., MacDessi S.J. (2018). Soft tissue balancing in total knee arthroplasty using sensor-guided assessment: Is there a learning curve?: Sensor assessment learning curve. ANZ J. Surg..

[8] Lakra A., Sarpong N.O., Jennings E.L., Grosso M.J., Cooper H.J., Shah R.P., Geller J.A. (2019). The Learning Curve by Operative Time for Soft Tissue Balancing in Total Knee Arthroplasty Using Electronic Sensor Technology. J. Arthroplast..

[9] Ghirardelli S., Bala A., Peretti G., Antonini G., Indelli P.F. (2019). Intraoperative Sensing Technology to Achieve Balance in Primary Total Knee Arthroplasty. JBJS Rev..

[10] Cho K.-J., Seon J.-K., Jang W.-Y., Park C.-G., Song E.-K. (2018). Objective quantification of ligament balancing using VERASENSE in measured resection and modified gap balance total knee arthroplasty. BMC Musculoskelet. Disord..

[11] Risitano S., Karamian B., Indelli P.F. (2017). Intraoperative load-sensing drives the level of constraint in primary total knee arthroplasty: Surgical technique and review of the literature. J. Clin. Orthop. Trauma.

[12] Walker P.S., Meere P.A., Bell C.P. (2014). Effects of surgical variables in balancing of total knee replacements using an instrumented tibial trial. Knee.

[13] Gustke K.A., Golladay G.J., Roche M.W., Elson L.C., Anderson C.R. (2017). A Targeted Approach to Ligament Balancing Using Kinetic Sensors. J. Arthroplast..

[14] Meere P.A., Schneider S.M., Walker P.S. (2016). Accuracy of Balancing at Total Knee Surgery Using an Instrumented Tibial Trial. J. Arthroplast..

[15] Cochetti A., Ghirardelli S., Iannotti F., Giardini P., Risitano S., Indelli P.F. (2020). Sensor-guided technology helps to reproduce medial pivot kinematics in total knee arthroplasty. J. Orthop. Surg..

[16] Bellemans J. (2011). Multiple Needle Puncturing: Balancing the Varus Knee. Orthopedics.

[17] Herschmiller T., Grosso M.J., Cunn G.J., Murtaugh T.S., Gardner T.R., Geller J.A. (2018). Step-wise medial collateral ligament needle puncturing in extension leads to a safe and predictable reduction in medial compartment pressure during TKA. Knee Surg. Sports Traumatol. Arthrosc..

[18] Luyckx T., Verstraete M., De Roo K., Van Der Straeten C., Victor J. (2016). High strains near femoral insertion site of the superficial medial collateral ligament of the Knee can explain the clinical failure pattern: 3D deformation analysis of the sMCL. J. Orthop. Res..

[19] Zapata G., Sanz-Pena I., Verstraete M., Walker P.S. (2019). Effects of femoral component placement on the balancing of a total knee at surgery. J. Biomech..

